# Change in trend of poliovirus strain affecting Pakistan

**DOI:** 10.1016/j.amsu.2022.104390

**Published:** 2022-08-23

**Authors:** Muhammad Hashir Nazir, Muhammad Ahmad, Muhammad Umer, Saleha Azeem

**Affiliations:** King Edward Medical University, 54000, Lahore, Pakistan

**Keywords:** WPV1, Wild Poliovirus type 1, cVDPV2, Circulating Vaccine Derived Poliovirus type 2, KP, Khyber Pakhtunkhwa

Dear editor,

The poliovirus, a species of Enterovirus C of the *Picornaviridae* family, is the primary cause of the highly contagious and crippling disease polio which primarily affects children under the age of five. It attacks the neurological system and results in paralysis or even death. Since it has no cure, immunisation is the only way to escape lifelong paralysis [1]. There exists wild poliovirus and vaccine-derived poliovirus. Since 1988 the number of cases of wild poliovirus has dropped by over 99%. Out of the 3 wild poliovirus strains (type 1, type 2, and type 3), type 2 was eradicated in 1999, and type 3 has not been reported since its last incidence in Nigeria in November 2012 [2]. As of 2022, wild poliovirus type 1(WPV1) affects only four countries: Pakistan (14 cases), Afghanistan (4 cases), Malawi (1 case) and Mozambique (4 cases). Moreover, attenuated oral poliovirus vaccine (OPV) derived poliovirus (cVDPV) is also posing a global threat with a total of 525 cases in 2022 to date [3]. Pakistan has shown a reemergence of polio cases from a historic low to a total of 14 cases in 2022 [4]. Pakistan is classified by the IHR (International health regulations) as a country with a potential risk of spreading wild poliovirus type 1 (WPV1) and circulating vaccine-derived poliovirus type 2 (cVDPV2) across international borders [5]. However, the strain which is seriously affecting Pakistan has changed. Poliovirus cases of the cVDPV2 strain rose from 22 cases in 2019 to 135 cases in 2020 and then declined from 8 cases in 2021 to zero in 2022 while the reported cases of WPV1 were 147 in 2019, 84 in 2020, 1 in 2021 and 14 in 2022 to date marking a rise in the cases of WPV1 with a simultaneous decrease in cVDPV2 cases [4]. This is represented in Fig. 1.

**Fig. 1:**
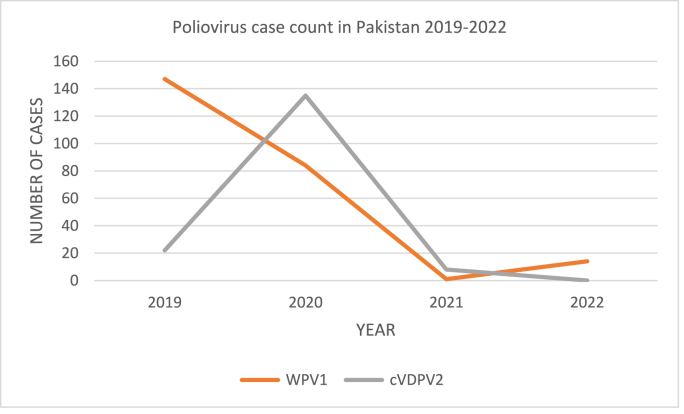
Poliovirus case count in Pakistan 2019–2022 [4].

In Pakistan, all the cases reported in 2022 are from the southern part of the northwestern province of Khyber-Pakhtunkhwa (KP). This province shares a border with Afghanistan, the only other polio-endemic country. This area includes the districts of North Waziristan and Laki Marwat with case counts of 13 and 1 in 2022 respectively [6]. In 2020, 22 cases were recorded in Khyber-Pakhtunkhwa (KP), while no wild poliovirus cases were recorded in the province in 2021. The environmental samples revealed the presence of wild poliovirus in southern KP [7]. The rate of vaccine refusal is high in these areas, considering the conservative lifestyle of inhabitants and the high illiteracy rate in the region which poses difficulties for the polio vaccination teams and has increased the number of children missed in the campaigns. In Pakistan's fight against the poliovirus, the cross-border movement of Afghan refugees in some areas of Waziristan is of great concern because there are 3.5 million unvaccinated children under five in Afghanistan [8]. The cases reported in Afghanistan in 2021 were all of the WPV1 strain which can explain the 14 WPV1 cases in Pakistan in 2022 [9]. Every year Pakistan conducts a nationwide polio vaccination campaign through more than 300,000 trained polio workers and vaccinates about 40 million children under the age of 5. This campaign continued unabated through 2021 and 2022 but there are important variations between provinces and districts [10]. In conclusion, Pakistan has been successful in its fight against cVDPV2 but now efforts are needed to curb wild poliovirus type 1 (WPV1). We recommend introducing social mobilizers, keeping political unrest from affecting immunisation campaigns and preventing the success story from turning into a failed endeavour.

## Ethical approval

N/A.

## Sources of funding

No source of funding to declare.

## Author contribution

Muhammad Hashir Nazir and Muhammad Ahmad: Performed data analysis and participated in writing the letter. Muhammad Umer: Participated in data analysis. Saleha Azeem: Reviewing the letter.

## Registration of research studies

Name of the registry:

N/A.

Unique Identifying number or registration ID:

N/A.

Hyperlink to your specific registration (must be publicly accessible and will be checked): N/A.

## Guarantor

Muhammad Ahmad.

Muhammad Hashir Nazir.

## Consent

N/A.

## Declaration of competing interest

No conflict of interest to declare.
